# Enhancing Red Table Grape Coloration Using Tsikoudia: A Novel and Sustainable Approach

**DOI:** 10.3390/plants13192689

**Published:** 2024-09-25

**Authors:** Emmanouil Kontaxakis, Dimitrios Lydakis, Ioannis Fisarakis

**Affiliations:** Department of Agriculture, School of Agricultural Sciences, Hellenic Mediterranean University, P.O. BOX 1939, GR 71410 Heraklion, Greece; dlydakis@hmu.gr (D.L.); fisar@hmu.gr (I.F.)

**Keywords:** Crimson Seedless, Red Globe, anthocyanin, ABA, ethephon

## Abstract

Achieving optimal coloration in red table grapes, especially in warm-climate regions, presents significant challenges due to high temperatures that inhibit anthocyanin biosynthesis. Conventional methods to enhance grape coloration, including the use of abscisic acid (ABA), ethephon, foliar nutrient supplementation, and viticultural practices like cluster trimming and girdling, have limitations related to cost, regulatory restrictions, and potential adverse effects on grapes quality. This study proposes the application of tsikoudia, a traditional Greek alcoholic beverage, as a novel, sustainable, and cost-effective alternative to conventional practices. Tsikoudia, applied during the veraison stage, significantly improved the coloration of ‘Crimson Seedless’ and ‘Red Globe’ grapes by enhancing anthocyanin accumulation and altering color parameters. Specifically, lightness (L*), chroma (C*), and hue angle (*h*), measured using the CIE-Lab color system, were reduced, while the Color Index for Red Grapes (CIRG) was increased. Additionally, total anthocyanin content, determined through spectrophotometric analysis, also showed an increase. These changes indicate a more intense red coloration. This research highlights the effectiveness of tsikoudia in improving grape coloration and contributes to the development of more sustainable viticultural practices.

## 1. Introduction

Table grape cultivation is a significant global economic activity, substantially contributing to the agricultural industry [1]. Among the various cultivars, red table grapes hold particular consumer demand due to their aesthetic attraction [2] and potential health benefits associated with their rich anthocyanin content [3]. These grapes’ bright red or purple coloration is crucial to their desirability. However, achieving optimal coloration in red table grapes can be challenging, mainly when grown in warm-climate regions [4,5,6].

The coloration of berries mainly arises from the synthesis and accumulation of anthocyanins within the skin of the grape berry. This process occurs through the flavonoid biosynthetic pathway and is influenced by various factors [7]. Climatic conditions, alongside numerous genetic, cultivation, and environmental factors, play a critical role in regulating the synthesis of anthocyanins [8,9,10]. However, elevated temperatures have a negative influence on anthocyanin accumulation [11], especially high night temperatures that inhibit anthocyanin biosynthesis due to lower expression levels of anthocyanin biosynthetic genes and lower activities of anthocyanin biosynthetic enzymes [12]. On the contrary, low temperatures at night were found to accelerate anthocyanin biosynthesis [13].

Efforts to enhance the coloration of red table grapes have led to the exploration of various techniques and treatments. These include applying abscisic acid and ethephon, using specialized nutrient products to aid grape maturation, and implementing viticultural practices like cluster trimming and girdling. 

Abscisic acid (ABA) is a phytohormone involved in several plant physiological processes, including anthocyanin accumulation and grape berry coloration during ripening. Nevertheless, since endogenous ABA levels are minimal at the preveraison stage [14], appropriate application of exogenous ABA has been found to improve the coloration of color grapes [15,16,17,18,19]. However, the cost of ABA application can be prohibitive for some growers.

Ethephon, a chemical precursor of ethylene, is a plant growth regulator with systemic properties, which is used mainly to promote the preharvest ripening of several products [20]. Several studies report its positive effect on the concentration of anthocyanins and the improvement of red grapes coloration [21,22,23]. However, its use is restricted in some viticultural regions due to regulatory concerns and challenges associated with elevated residue levels in grapes during harvest [24].

Numerous foliar nutrient products, rich in potassium and other essential elements, have been investigated for their potential to impact grape color development [25,26]. These products are specifically formulated to support grape maturation and may also enhance the accumulation of anthocyanins, which contribute to grape coloration. However, their efficacy may not be consistent and reliable, as the ripening process appears to play a more significant role in determining berry color than nutrient applications [27].

Certain viticultural practices, including cluster trimming and girdling at the veraison stage, have been identified as effective methods for enhancing grape color [21,28,29]. However, these methods are inefficient and require a lot of labor and time. Additionally, cluster trimming leads to lower yields [30], and girdling can potentially trigger wood-related diseases due to bark removal [31,32,33].

Recent studies have explored novel approaches, such as using blue- and red-light irradiation during the night to increase anthocyanin and ABA concentrations in grapes [34,35]. However, the practicality and cost-effectiveness of implementing LED lighting systems in vineyards remain subjects of consideration.

Finally, significant improvement in the coloration of red grapes has been observed by applying ethanol or methanol via spraying on the grapes preharvest, particularly at the veraison stage [21,36,37]. 

Considering these various approaches and the potential benefits of ethanol or methanol application on grape coloration, this study examines the effects of a traditional Greek alcoholic beverage known as “tsikoudia” on the coloration of red grapes. Tsikoudia offers several advantages, including ease of application, cost-effectiveness, absence of residue in grapes, and the possibility for grape growers to produce it themselves through the distillation of fermented grape marc. Furthermore, tsikoudia is a natural, environmentally friendly product that aligns with organic farming principles.

This study aims to explore the potential of tsikoudia as a novel and sustainable approach to enhance the coloration of red table grapes, providing insights into its efficacy and practicality in viticultural applications.

## 2. Results and Discussion

### 2.1. Physicochemical Characteristics of Grapes

According to the results, none of the treatments applied to enhance grape coloration, including tsikoudia, ethrel, abscisic acid (ABA), madurel, and cluster trimming, had any significant positive or negative effects on most of the physical characteristics of both studied grape cultivars (Table 1 and Table 2). 

This outcome is important because such treatments can sometimes lead to significant disadvantages, including unintended adverse effects on grape quality, such as berry shatter [38] and berry softening, mainly when ABA or ethephon are used [22,39,40]. However, the conditions under which these negative consequences occur should be investigated, as in many cases, the quality characteristics of grapes, as seen in this study, are not affected [4,41] or even improve [42].

However, in both grape cultivars, it was observed that cluster trimming resulted in a significant reduction in the length of the bunches when compared to both the other treatment groups and the control group (Table 1 and Table 2). This decrease in size can be ascribed to the removal of approximately one-quarter of the bunch tail during the veraison stage within the framework of this specific handling procedure [30].

Moreover, significant effects were observed on the total soluble solids, titratable acidity of the must, and the maturity index. In the ‘Crimson Seedless’ cultivar, the application of ethrel in the first year and all treatments in the second year led to an increase in the sugar content in the must (Table 1 and Table 2). Similarly, treatments with tsikoudia, ABA, and madurel in the first year and all treatments in the second year reduced the titratable acidity of the must. Consequently, the maturity index at harvest was significantly higher in the treatments with tsikoudia, ethrel, and ABA in the first year and all treatments in the second year, compared to the control group.

In the ‘Red Globe’ cultivar, the application of tsikoudia and ethrel led to an increase in sugar content, while all treatments resulted in a reduction in the acidity of the must, which was consistently observed over the two years of the study (Table 2). Consequently, the maturity index at harvest was significantly higher in all treatments compared to the control group for both years of the study.

Based on the above results, it is evident that the treatments under investigation, mainly those involving tsikoudia and ethrel, have enhanced the ripening of grapes. This effect can be considered favorable when applied to early grape cultivars and when early ripening is desired. However, this influence may have adverse consequences in the case of late grape cultivars and when delayed ripening is desired [43,44]. 

The results demonstrate that the application of tsikoudia had similar effects on increasing total soluble solids and reducing titratable acidity to the conventional methods of applying ABA, ethephon, methanol, and ethanol [21,36,45,46].

### 2.2. Coloration of Grapes

The color parameters lightness (L*), chroma (C*), and hue angle (*h*) of the berries were evaluated using the CIE-Lab color system and a Minolta Chroma Meter. The findings indicate that all the treatments significantly influenced the coloration of both examined grape cultivars within both years of the study.

**Figure 1:**
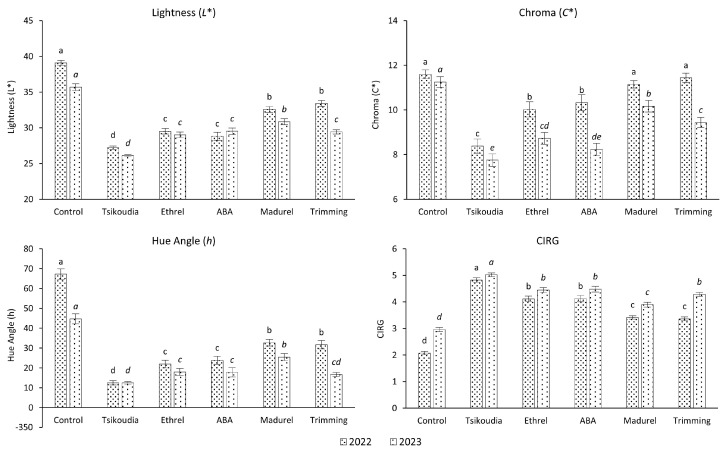
Effect of treatments on the coloration of ‘Crimson Seedless’ grapes, expressed as CIE-Lab color parameters lightness (L*), chroma (C*), hue angle (*h*), and Color Index for Red Grapes (CIRG). The different letters for each year (in italics for the 2023) indicate significant differences according to Duncan’s multiple range test (*p* ≤ 0.05) among the treatments. Error bars represent the standard errors of the means.

Significantly improved coloration was achieved in the ‘Crimson Seedless’ cultivar for both years through tsikoudia treatment. Specifically, it had a significant impact on reducing the lightness parameter (resulting in a darker color), reducing the chroma parameter (resulting in a denser color), and reducing the hue angle parameter (shifting towards 0° where the red color is located) compared to the rest treatments and the control. Furthermore, the tsikoudia treatment had significantly higher scores on the CIRG index, indicating a superior red coloration. The treatments with ethrel and ABA were less effective than the tsikoudia treatment. However, they significantly enhanced red coloration (CIRG) compared to the madurel and cluster trimming treatments, which also showed improvements relative to the control group (Figure 1).

**Figure 2:**
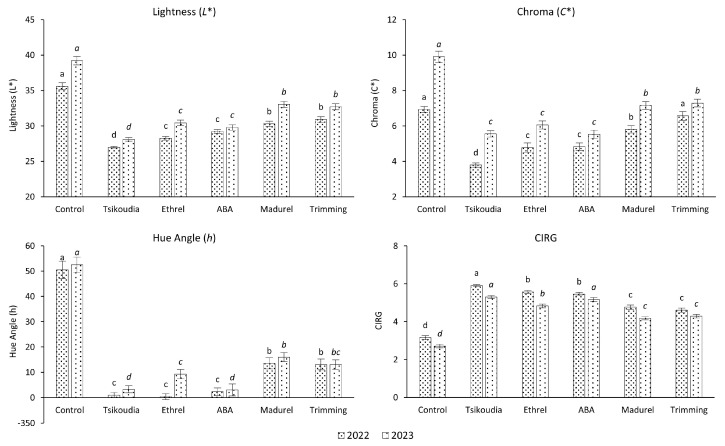
Effect of treatments on the coloration of ‘Red Globe’ grapes, expressed as CIE-Lab color parameters lightness (L*), chroma (C*), hue angle (*h*), and Color Index for Red Grapes (CIRG). The different letters for each year (in italics for the 2023) indicate significant differences according to Duncan’s multiple range test (*p* ≤ 0.05) among the treatments. Error bars represent the standard errors of the means.

The results were similar for the ‘Red Globe’ cultivar, as the application of tsikoudia significantly enhanced the red coloration compared to all other treatments, including the control group in the first year, as well as in comparison to all treatments except for the ABA treatment in the second year, which had a similar positive impact. Like the ‘Crimson Seedless’ cultivar, all treatments significantly improved red coloration, although the treatments with madurel and cluster trimming were not as effective as the other treatments (Figure 2).

The results indicate that the tsikoudia treatment improved the coloration of ‘Crimson Seedless’ and ‘Red Globe’ grapes with effectiveness comparable to, or even better than, established methods known to enhance color in red grape cultivars. These established methods include the application of ABA [38,40], ethephon [6,22], ethanol or methanol [21,47], foliar nutrient products, and cluster trimming [48], typically used when grapes fail to achieve the desired color coverage and intensity.

### 2.3. Total Anthocyanin Content

**Figure 3:**
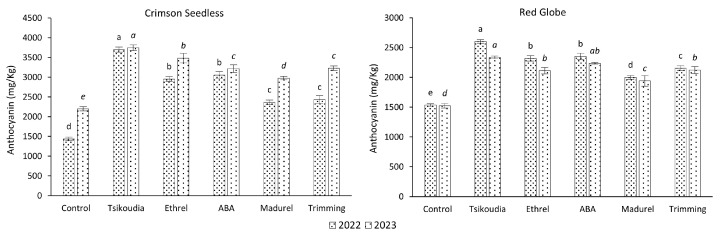
Effect of treatments on the total anthocyanin content in ‘Crimson Seedless’ and ‘Red Globe’ grape skins. The different letters for each year (in italics for the 2023) indicate significant differences according to Duncan’s multiple range test (*p* ≤ 0.05) among the treatments. Error bars represent the standard errors of the means.

Based on the determination of total anthocyanin content, all treatments significantly increased the anthocyanin content in grape skins over both years and for both cultivars studied. However, the most significant increase was observed in the treatment involving the application of tsikoudia, with results corresponding to the measurements of grape berry color. Among the remaining treatments, the best results were noted for applications with ethrel or ABA. Treatments with madurel and cluster trimming had a lesser impact than the other treatments; nevertheless, they still significantly increased anthocyanin content and improved grape coloration compared to the control (Figure 3).

The observed increase in anthocyanin accumulation following tsikoudia application aligns with previous studies demonstrating the positive impact of ethanol on anthocyanin biosynthesis and grape color enhancement [37,49]. The effectiveness of tsikoudia in improving grape coloration is likely due to ethanol, its primary component, which is known to induce stress responses that promote anthocyanin production. Ethanol acts as a signaling molecule, activating the phenylpropanoid pathway, which is involved in the biosynthesis of anthocyanins. This pathway is typically triggered in response to various abiotic stresses, leading to the production of phenolic compounds, including flavonoids and anthocyanins [50,51]. 

However, the intensity of the results was remarkable, showing better coloration than established methods including ABA, ethephon, ethanol, methanol, foliar nutrient products, and cluster trimming. This suggests that the positive effect of tsikoudia on grape coloration can be attributed not only to its main component, ethanol, but also to other constituents identified through NMR analysis, such as methanol, ethyl acetate, ethyl lactate, acetaldehyde, 2-methyl-3-propanol, 3-methyl-3-butanol, propanol-1, and phenylethanol, common constituents in tsikoudia and similar spirits [52]. It is speculated that these components, either individually or in combination, enhanced grape coloration, providing tsikoudia with an advantage over ethanol alone. However, to date, there are no studies confirming the impact of these components and their concentration on grape coloration, except for methanol, which has been shown to have a positive effect [21,36]. Despite the low concentration of methanol in the dilution, its contribution and that of the other components warrant further investigation. Future studies should explore the effects of each component and their combinations on red grape coloration. The effectiveness of tsikoudia is essential, given that it offers several advantages, including ease of application, cost-effectiveness, lack of residue in grapes, and the potential for grape growers to produce it themselves through the distillation of fermented grape marc.

## 3. Materials and Methods

### 3.1. Growing Conditions and Experimental Design

The research was conducted over two years (2022 and 2023) in two vineyards in the viticultural region of Archanes (Crete, Greece), selected for their historical and recurrent challenges with grape coloration. The ‘Crimson Seedless’ vineyard was 15 years old, with grapevines grafted onto ‘41B’ rootstock, while the ‘Red Globe’ vineyard was 20 years old, with grapevines grafted onto ‘1013P’ rootstock. Both cultivars were trained using a V-type trellis system.

This study evaluated the impact of tsikoudia application during the veraison stage on grape coloration, using a total of six treatments. A control group of nontreated grapevines was included, along with grapevines treated with standard commercial methods, such as ABA application, ethephon, nutrient supplementation (Madurel), and cluster trimming, which also served as control treatments to establish a baseline for comparison.

A randomized complete block design (RCBD) was used, with five replicates and three vines per plot. All applications were carried out twice during the veraison stage. The first application took place when 5–20% of the berries had changed color, followed by the second application one week later. Applications were carried out early in the morning to avoid high temperatures and direct sun exposure, which can cause rapid evaporation and significantly reduce the absorption of the spray solution. The mean air temperature during the application was 24 to 25 °C for both years. The spraying applications were carried out using an electrostatic backpack sprayer [53].

Tsikoudia (a traditional Greek alcoholic beverage) with an alcohol content of 38.37%, distilled from fermented grape marc, was supplied by a local distiller. The composition of tsikoudia was analyzed using an NMR spectroscopic analytical technique [54], revealing the following components: alcohol: 38.37% *v*/*v*, methanol: 1.2784 g L^−1^, ethyl acetate: 0.5289 g L^−1^, ethyl lactate: 0.2743 g L^−1^, acetaldehyde: 0.0294 g L^−1^, 2-methyl-3-propanol: 0.1806 g L^−1^, 3-methyl-3-butanol: 0.6287 g L^−1^, propanol-1: 0.1784 g L^−1^, and phenylethanol: 1.2216 g L^−1^.

The tsikoudia was diluted with distilled water to attain a 30% alcohol content before being utilized for spraying. For the conventional treatments, concentrations were selected according to the manufacturers’ recommendations. Specifically, ethephon (Ethrel^®^ Top SL, 48 SL, Bayer, Leverkusen, Germany) was applied at a concentration of 480 mg L^−1^. ABA (Protone SL, Valent BioSciences Co., Libertyville, IL, USA) was applied at a concentration of 400 mg L^−1^. Madurel (Agriside CropCare, Giannitsa, Greece), a nutrient supplement, was used at a concentration of 5 mL L^−1^. For the control group, grapes were sprayed with water. Ethoxylated isodecyl alcohol (Haiten Plus 15 SL, Phytorgan SA, Athens, Greece) was incorporated at a concentration of 45 mg L^−1^ in all treatments, including the control, to improve the wettability and distribution of the spray coating on the grapes. The cluster trimming (CT) treatment entailed removing approximately one-quarter of the bunch tail at the veraison stage. This procedure decreased the overall size of the clusters and gave them a more rounded shape.

### 3.2. Evaluation of Physicochemical Characteristics of Grapes

The physical and chemical characteristics of grapes were assessed at harvest time, which was conducted on the same day for all treatments to facilitate their comparative evaluation. The determination of the harvest day was based on the total soluble solids (TSS > 16 °Brix). For the measurements, five bunches were randomly selected for each treatment and cultivar from the central grapevine of each plot, with the others designated as buffer zones.

The dimensions of the bunches were measured with a digital caliper, and their weight was determined using a precision laboratory scale. The average values of ten randomly selected berries from each bunch were utilized to evaluate the berries’ physical characteristics. Berry shatter was assessed by counting the number of detached berries from grape bunches attached by their peduncles on a vibrating arm of a laboratory shaker (Big Bill, Thermolyne, Dubuque, IA, USA), following 30 s of vibration at a speed of 500 rpm, and the results were expressed as a percentage of the total number of berries [55]. Total soluble solids (°Brix) were estimated using a digital Brix-Acidity Meter PAL-BX|ACID (Atago, Tokyo, Japan). Titrable acidity (g H_2_Ta L^−1^) was determined through titration with NaOH 0.1 N (OIV-MA-AS313-01). The maturation index (ratio) was calculated as the sugar-to-acid ratio.

### 3.3. Grape Coloring Evaluation

The color parameters lightness (L*), chroma (C*), and hue angle (*h*) of the berries were evaluated using the CIE-Lab color system (CIE, 2007), with a Minolta Chroma Meter (Konica Minolta CR-400, Tokyo, Japan). 

Average values of twenty randomly selected berries were used for each of the five bunches per treatment. Hue angle values below 360° (red color on hue-circle = 0° = 360°) were transformed to negative values (e.g., 360° − 350° = −10°) for statistical analysis purposes and mean values calculation [56,57]. Color Index for Red Grapes (CIRG) was also evaluated using the formula [CIRG = (180° − *h*)/(C* + L*)] [57].

### 3.4. Determination of Total Anthocyanin Content

The total anthocyanin content of berry skins was determined using the following method [58]. The skins were peeled from the berries, and a known weight portion was suitably diluted to achieve an absorbance between 0.3 and 0.7 in the extract. The absorbance was measured at 537 nm using a Shimadzu UV-1800 Spectrophotometer (Shimadzu, Tokyo, Japan). The total anthocyanin content was then estimated using a calibration curve, which was constructed by plotting the absorbance (A537) of known concentrations of Oenin chloride (Extrasynthese, Genay, France) ranging from 0.03 to 0.5 mg mL^−1^, with an R² value of 0.99. The results were expressed as mg of oenin equivalents (OE) per gram of fresh weight (FW).

### 3.5. Statistical Analysis

One-way ANOVA was conducted utilizing the Statistical Package for the Social Sciences (IBM SPSS Statistics, version 29). Microsoft Excel 365 (Microsoft, Redmond, WA, USA) was employed for statistical analysis and graphical representation. Treatment means are presented in the graphics and tables. Significance levels were indicated by symbols: ns = *p* > 0.05; * = *p* ≤ 0.05; ** = *p* ≤ 0.01; *** = *p* ≤ 0.001. Distinct letters were used to signify significant differences among the treatments, as determined by Duncan’s multiple range test (*p* ≤ 0.05).

## 4. Conclusions

This study evaluated the efficacy of tsikoudia, a traditional Greek alcoholic beverage, as a novel and sustainable method to enhance red table grape coloration. Tsikoudia, applied twice during the veraison stage, significantly improved the coloration of ‘Crimson Seedless’ and ‘Red Globe’ grapes by reducing lightness, chroma, and hue angle color parameters, while increasing anthocyanin content in grape skins. The treatment proved to be more effective than conventional methods and did not negatively affect grape quality. It also increased total soluble solids and reduced titratable acidity, leading to a higher maturity index at harvest.

In conclusion, this study provides robust evidence supporting the use of tsikoudia as an effective, sustainable, and cost-efficient method for enhancing red table grape coloration. The advantages of tsikoudia include its ease of application, lack of residual chemicals, and the potential for grape growers to produce it through the distillation of fermented grape marc. These benefits make tsikoudia a promising alternative to conventional chemical treatments, aligning with organic farming principles and contributing to sustainable viticulture. Further research is needed to explore the effects of individual components in tsikoudia, their concentrations, and their combinations on red grape coloration.

## Figures and Tables

**Table 1:** Effect of treatments on the physical and chemical characteristics of ‘Crimson Seedless’ grapes. Values are treatments means ± standard deviation; significance level (Sig.): ns = *p* > 0.05; * = *p* ≤ 0.05; ** = *p* ≤ 0.01; *** = *p* ≤ 0.001. The different letters among the treatments indicate significant differences according to Duncan’s multiple range test (*p* ≤ 0.05).

		Control		Tsikoudia		Ethrel		ABA		Madurel		Cluster Trimming		Sig.
Bunch Length (cm)	2022	25.6 ^a^	±2.9	27.5 ^a^	±1.4	24.8 ^a^	±3.0	23.8 ^a^	±3.6	26.3 ^a^	±5.3	16.1 ^b^	±3.4	***
	2023	19.4 ^a^	±3.6	20.1 ^a^	±2.0	21.3 ^a^	±0.5	20.8 ^a^	±2.2	20.2 ^a^	±1.2	15.1 ^b^	±0.9	***
Bunch Width (cm)	2022	15.5 ^a^	±1.1	16.2 ^a^	±3.5	15.1 ^a^	±4.3	17.9 ^a^	±3.4	16.8 ^a^	±1.1	15.6 ^a^	±2.6	ns
	2023	18.0 ^a^	±3.8	15.5 ^a^	±3.5	16.6 ^a^	±1.8	16.4 ^a^	±3.1	16.9 ^a^	±2.8	17.4 ^a^	±1.8	ns
Bunch Weight (g)	2022	410.8 ^a^	±86.5	519.6 ^a^	±138.6	517.8 ^a^	±171.2	569.0 ^a^	±167.6	570.5 ^a^	±72.7	380.7 ^a^	±116.1	ns
	2023	543.5 ^a^	±212.2	577.6 ^a^	±127.6	678.9 ^a^	±158.9	624.5 ^a^	±262.8	598.1 ^a^	±123.4	529.4 ^a^	±138.2	ns
Rachis Length (cm)	2022	25.1 ^a^	±3.1	26.3 ^a^	±1.5	24.7 ^a^	±4.5	23.6 ^a^	±4.3	25.2 ^a^	±5.3	15.1 ^b^	±3.2	***
	2023	17.4 ^a^	±3.9	19.7 ^a^	±1.9	20.3 ^a^	±0.5	19.7 ^a^	±2.5	19.4 ^a^	±1.0	13.8 ^b^	±0.6	***
Rachis Weight (g)	2022	15.9 ^ab^	±1.6	17.4 ^a^	±0.7	15.8 ^ab^	±7.1	17.1 ^ab^	±7.0	17.8 ^a^	±3.1	10.8 ^b^	±2.3	ns
	2023	11.5 ^a^	±1.6	10.7 ^a^	±2.0	12.2 ^a^	±3.6	10.4 ^a^	±3.5	10.2 ^a^	±2.1	9.0 ^a^	±2.5	ns
Berry Shatter (%)	2022	0.7 ^a^	±0.7	1.0 ^a^	±1.9	2.7 ^a^	±2.3	2.3 ^a^	±1.1	1.9 ^a^	±2.4	2.8 ^a^	±1.8	ns
	2023	1.8 ^a^	±1.7	2.9 ^a^	±2.3	1.4 ^a^	±0.7	1.9 ^a^	±2.0	3.0 ^a^	±2.2	2.0 ^a^	±2.8	ns
Berry Weight (g)	2022	3.8 ^a^	±0.7	3.9 ^a^	±0.6	3.9 ^a^	±0.7	3.7 ^a^	±0.5	3.9 ^a^	±0.2	4.2 ^a^	±0.5	ns
	2023	3.5 ^b^	±0.1	3.8 ^ab^	±0.7	3.8 ^ab^	±0.4	3.5 ^b^	±0.6	4.0 ^ab^	±0.5	4.4 ^a^	±0.4	ns
Berries Amount	2022	107.2 ^ab^	±15.5	127.2 ^ab^	±22.5	131.0 ^ab^	±45.6	151.4 ^a^	±57.1	142.2 ^a^	±16.9	88.2 ^b^	±18.7	ns
	2023	152.6 ^a^	±61.9	154.0 ^a^	±37.1	176.4 ^a^	±35.9	170.2 ^a^	±54.6	147.4 ^a^	±24.0	117.2 ^a^	±34.1	ns
Small BerriesAmount	2022	4.4 ^a^	±3.1	6.0 ^a^	±3.3	2.8 ^a^	±3.1	7.6 ^a^	±6.3	8.2 ^a^	±7.7	2.2 ^a^	±1.3	ns
	2023	4.4 ^a^	±3.1	0.4 ^b^	±0.6	2.6 ^ab^	±4.2	0.6 ^b^	±0.9	2.2 ^ab^	±2.8	0.8 ^b^	±1.1	ns
Total Soluble Solids (°Brix)	2022	18.0 ^b^	±0.9	19.0 ^ab^	±1.7	19.7 ^a^	±0.4	18.7 ^ab^	±0.5	18.3 ^b^	±0.3	17.8 ^b^	±0.8	*
	2023	17.0 ^b^	±0.4	18.9 ^a^	±1.2	18.6 ^a^	±0.5	18.7 ^a^	±1.1	18.1 ^a^	±0.9	18.1 ^a^	±0.3	*
Titratable Acidity (g H_2_ Ta L^−1^)	2022	6.0 ^a^	±0.5	4.9 ^b^	±0.5	5.5 ^ab^	±0.6	5.0 ^b^	±0.2	5.3 ^b^	±0.4	5.4 ^ab^	±0.5	**
	2023	7.7 ^a^	±0.4	6.7 ^b^	±0.3	6.6 ^b^	±0.1	6.4 ^b^	±0.7	6.7 ^b^	±0.3	6.7 ^b^	±0.8	**
Maturity Index	2022	30.0 ^c^	±3.7	39.5 ^a^	±6.3	36.5 ^ab^	±4.2	37.5 ^ab^	±2.4	34.7 ^abc^	±2.3	33.1 ^bc^	±3.0	*
	2023	22.0 ^b^	±1.9	28.2 ^a^	±2.1	28.2 ^a^	±1.3	30.0 ^a^	±5.4	27.0 ^a^	±2.2	27.2 ^a^	±3.3	**
pH	2022	3.2 ^a^	±0.1	3.6 ^a^	±0.1	3.2 ^a^	±0.1	3.9 ^a^	±0.1	3.2 ^a^	±0.0	3.2 ^a^	±0.1	ns
	2023	3.6 ^a^	±0.1	3.6 ^a^	±0.1	3.6 ^a^	±0.1	3.6 ^a^	±0.0	3.5 ^a^	±0.0	3.5 ^a^	±0.0	ns

**Table 2:** Effect of treatments on the physical and chemical characteristics of ‘Red Globe’ grapes. Values are treatments means ± standard deviation; significance level (Sig.): ns = *p* > 0.05; * = *p* ≤ 0.05; ** = *p* ≤ 0.01; *** = *p* ≤ 0.001. The different letters among the treatments indicate significant differences according to Duncan’s multiple range test (*p* ≤ 0.05).

		Control		Tsikoudia		Ethrel		ABA		Madurel		Cluster Trimming		Sig.
Bunch Length (cm)	2022	31.1 ^a^	±1.3	31.0 ^a^	±2.8	33.9 ^a^	±3.2	31.9 ^a^	±4.2	33.4 ^a^	±4.3	25.6 ^b^	±3.5	**
	2023	27.4 ^a^	±3.3	27.2 ^a^	±5.6	27.1 ^a^	±3.6	28.1 ^a^	±7.1	27.2 ^a^	±2.9	19.4 ^a^	±1.0	*
Bunch Width (cm)	2022	19.4 ^a^	±4.2	19.2 ^a^	±1.9	19.0 ^a^	±3.5	16.7 ^a^	±2.8	16.1 ^a^	±1.1	16.2 ^a^	±3.0	ns
	2023	18.4 ^a^	±2.8	19.6 ^a^	±1.3	17.3 ^a^	±1.7	17.0 ^a^	±1.6	18.0 ^a^	±2.7	19.5 ^a^	±2.6	ns
Bunch Weight (g)	2022	899.8 ^a^	±339.3	973.4 ^a^	±144.5	973.5 ^a^	±213.6	812.2 ^a^	±196.2	754.1 ^a^	±81.2	692.3 ^a^	±230.8	ns
	2023	1011.8 ^a^	±163.0	1167.8 ^a^	±95.4	971.5 ^a^	±339.6	965.4 ^a^	±154.7	1006.4 ^a^	±264.5	863.5 ^a^	±157.8	ns
Rachis Length (cm)	2022	29.9 ^a^	±1.3	29.8 ^a^	±2.6	32.3 ^a^	±3.1	30.4 ^a^	±4.7	31.5 ^a^	±4.1	24.5 ^b^	±3.5	*
	2023	26.0 ^a^	±3.0	25.3 ^a^	±5.6	26.0 ^a^	±3.9	26.6 ^a^	±6.3	25.2 ^a^	±2.9	18.5 ^a^	±0.9	*
Rachis Weight (g)	2022	19.2 ^a^	±0.6	20.9 ^a^	±1.2	20.3 ^a^	±2.1	19.4 ^a^	±1.8	20.9 ^a^	±2.7	16.1 ^b^	±2.5	**
	2023	17.0 ^a^	±2.3	17.7 ^a^	±4.8	17.7 ^a^	±1.7	17.7 ^a^	±3.8	17.6 ^a^	±1.8	14.7 ^a^	±0.7	ns
Berry Shatter (%)	2022	0.7 ^a^	±1.0	0.4 ^a^	±0.5	0.3 ^a^	±0.7	0.6 ^a^	±0.9	0.5 ^a^	±0.7	1.0 ^a^	±1.5	ns
	2023	1.3 ^a^	±1.3	1.0 ^a^	±1.0	0.0 ^a^	±0.0	1.4 ^a^	±1.5	0.6 ^a^	±0.6	0.6 ^a^	±0.8	ns
Berry Weight (g)	2022	9.0 ^a^	±1.6	10.1 ^a^	±1.0	9.4 ^a^	±1.7	8.7 ^a^	±1.7	9.6 ^a^	±1.2	9.3 ^a^	±1.6	ns
	2023	11.3 ^a^	±2.6	11.7 ^a^	±1.0	10.0 ^a^	±0.6	11.7 ^a^	±0.5	11.1 ^a^	±0.8	9.8 ^a^	±1.7	ns
Berries Amount	2022	99.4 ^a^	±33.9	98.4 ^a^	±21.0	104.0 ^a^	±32.1	91.2 ^a^	±11.3	73.8 ^a^	±16.0	75.4 ^a^	±31.4	ns
	2023	89.8 ^a^	±13.0	103.4 ^a^	±10.9	97.0 ^a^	±32.3	75.0 ^a^	±19.3	88.4 ^a^	±19.2	91.4 ^a^	±14.4	ns
Small Berries Amount	2022	4.6 ^a^	±3.6	2.0 ^a^	±2.4	5.4 ^a^	±4.5	4.8 ^a^	±5.4	2.6 ^a^	±3.1	3.2 ^a^	±4.5	ns
	2023	2.8 ^a^	±2.6	1.2 ^a^	±0.8	6.4 ^a^	±7.6	1.4 ^a^	±1.9	1.2 ^a^	±1.3	3.4 ^a^	±5.5	ns
Total Soluble Solids (°Brix)	2022	13.0 ^b^	±0.3	15.1 ^a^	±1.0	15.4 ^a^	±1.6	14.4 ^ab^	±0.8	14.6 ^ab^	±0.7	14.5 ^ab^	±2.3	*
	2023	12.4 ^b^	±2.1	14.9 ^a^	±0.8	14.5 ^a^	±0.8	14.0 ^ab^	±1.3	13.4 ^ab^	±0.6	13.1 ^ab^	±1.2	*
Titratable Acidity (g H_2_ Ta L^−1^)	2022	6.5 ^a^	±0.4	4.9 ^b^	±0.7	4.1 ^b^	±0.3	4.4 ^b^	±0.6	4.6 ^b^	±0.7	5.0 ^b^	±0.8	***
	2023	6.2 ^a^	±0.4	5.0 ^b^	±0.6	5.1 ^b^	±0.8	5.2 ^b^	±0.6	5.0 ^b^	±0.7	5.0 ^b^	±0.7	*
Maturity Index	2022	19.9 ^b^	±1.5	31.4 ^a^	±4.3	37.4 ^a^	±5.7	33.4 ^a^	±6.9	32.6 ^a^	±5.4	30.3 ^a^	±10.9	**
	2023	20.0 ^b^	±3.3	30.2 ^a^	±5.6	28.9 ^a^	±4.4	27.5 ^a^	±5.7	27.6 ^a^	±5.6	26.6 ^a^	±4.0	*
pH	2022	3.6 ^a^	±0.1	3.6 ^a^	±0.1	3.6 ^a^	±0.1	3.5 ^a^	±0.0	3.5 ^a^	±0.0	3.5 ^a^	±0.1	ns
	2023	3.5 ^a^	±0.0	3.6 ^a^	±0.0	3.6 ^a^	±0.1	3.5 ^a^	±0.1	3.5 ^a^	±0.0	3.5 ^a^	±0.1	ns

## Data Availability

The original contributions presented in the study are included in the article, further inquiries can be directed to the corresponding author.
